# AICAR-Induced Activation of AMPK Inhibits TSH/SREBP-2/HMGCR Pathway in Liver

**DOI:** 10.1371/journal.pone.0124951

**Published:** 2015-05-01

**Authors:** Shudong Liu, Fei Jing, Chunxiao Yu, Ling Gao, Yejun Qin, Jiajun Zhao

**Affiliations:** 1 Department of Endocrinology, Provincial Hospital Affiliated to Shandong University, Jinan, China; 2 Central Laboratory, Provincial Hospital Affiliated to Shandong University, Jinan, China; 3 Institute of Endocrinology, Shandong Academy of Clinical Medicine, Jinan, China; 4 Department of Pathology, Provincial Hospital Affiliated to Shandong University, Jinan, China; 5 Department of Endocrinology, Shandong Rongjun General Hospital, Jinan, China; Boston University School of Medicine, UNITED STATES

## Abstract

Our previous study found that thyroid-stimulating hormone promoted sterol regulatory element-binding protein-2 (SREBP-2) expression and suppressed AMP-activated protein kinase (AMPK) activity in the liver, but it was unclear whether there was a direct link between TSH, AMPK and SREBP-2. Here, we demonstrate that the 5-aminoimidazole-4-carboxyamide ribonucleoside (AICAR)-induced activation of AMPK directly inhibited the expression of SREBP-2 and its target genes HMGCR and HMGCS, which are key enzymes in cholesterol biosynthesis, and suppressed the TSH-stimulated up-regulation of SREBP-2 in HepG2 cells; similar results were obtained in TSH receptor knockout mice. Furthermore, AMPK, an evolutionally conserved serine/threonine kinase, phosphorylated threonine residues in the precursor and nuclear forms of SREBP-2, and TSH interacted with AMPK to influence SREBP-2 phosphorylation. These findings may represent a molecular mechanism by which AMPK ameliorates the hepatic steatosis and hypercholesterolemia associated with high TSH levels in patients with subclinical hypothyroidism (SCH).

## Introduction

Thyroid-stimulating hormone is secreted by the pituitary and regulates thyroid growth and differentiation. However, recent studies have discovered that TSH is a tropic hormone that has multiple effects on metabolism, including cholesterol synthesis and glucose metabolism [1, 2]. Several epidemiological studies have indicated positive correlations between TSH and total cholesterol, triglycerides, and low-density lipoprotein cholesterol (LDL-c) [3–6]. Subclinical hypothyroidism (SCH), which is characterized by normal thyroid hormone levels and elevated TSH levels, is often accompanied by hypercholesterolemia and associated with cardiovascular disease [7]. Therefore, more studies are focusing on the relationship between TSH and hypercholesterolemia to explore novel approaches for preventing cardiovascular disease.

The liver is the major organ involved in cholesterol metabolism. Sterol regulatory element-binding protein-2 (SREBP-2) is synthesized as a precursor protein in the endoplasmic reticulum and subsequently undergoes sequential proteolytic cleavage to attain the N-terminal transcriptionally active form; the active form translocates into the nucleus to promote the expression of target genes involved in cholesterol biosynthesis and uptake in the liver, such as 3-hydroxy-3-methylglutaryl-CoA synthase (HMGCS), 3-hydroxy-3-methylglutaryl-CoA reductase (HMGCR) and low-density lipoprotein receptor (LDLR) [8,9]. Liver-specific SREBP-2 transgenic mice exhibit a significant increase in the rate of cholesterol synthesis and elevated expression of HMGCS (13-fold), HMGCR (75-fold) and LDLR (5.8-fold) [10]. Our previous research demonstrated that TSH upregulates the expression of HMGCR, a rate-limiting enzyme in liver cholesterol synthesis, via the cAMP/PKA/CREB pathway [1]. Furthermore, Min HK et al. showed that the accumulation of triglycerides and free cholesterol in nonalcoholic fatty liver disease (NAFLD) was accompanied by the inhibition of AMPK activity and increased expression of SREBP-2 and HMGCR [11]. Recently, evidence has suggested that TSH levels are closely associated with NAFLD [12–14]. Therefore, these findings suggest a possible new role for TSH in hepatic steatosis and dyslipidemia. We found that TSH increases mRNA expression of SREBP-2 in liver cells (unpublished), but the precise molecular pathway remains unclear.

AMP-activated protein kinase (AMPK) is a crucial cellular energy sensor that is activated by phosphorylation at Thr^172^ in the alpha subunit in response to various metabolic stressors, and AMPK is a major regulator of glucose and lipid metabolism that phosphorylates and inactivates numerous metabolic enzymes, including glycogen synthase, acetyl-CoA carboxylase (ACC), HMGCR, and CREB-regulated transcription coactivator 2 (CRTC2) [15]. Recently, AMPK has attracted more interest as a pharmacological target for therapeutic intervention in metabolic disorders [16–19]. Chronic activation of AMPK leads to a compensatory increase in SREBP-2 and HMGCR expression in the liver of a new transgenic mouse model with liver-specific expression of constitutively active (CA)-AMPK-α1 [20], but in adipose tissue, SREBP-2 and HMGCR mRNA expression was down-regulated [21]. A recent study demonstrated that AMPK interacts with and phosphorylates SREBP-1 and SREBP-2, thereby inhibiting SREBP activation by impeding its proteolytic maturation and nuclear translocation [22].

However, it remains unclear whether there is a direct link between TSH, AMPK and SREBP-2. Therefore, we aimed to investigate whether AMPK plays an active role in regulating hepatic cholesterol metabolism by modulating SREBP-2 and whether AMPK affects the TSH-induced up-regulation of SREBP-2 in the liver; these data may provide a basis for the future clinical treatment of hypercholesterolemia associated with TSH.

## Materials and Methods

### Reagents and antibodies

5-Aminoimidazole-4-carboxyamide ribonucleoside (AICAR) was from Toronto Research Chemicals (Downsview, ON, Canada). Adenosine 5´-monophosphate sodium salt (5´AMP), bTSH, threo-1, 4-Dimercapto-2, 3-butanediol (DTT) and were purchased from Sigma (St. Louis, MO). RIPA lysis buffer and bicinchoninic acid (BCA) protein assay kits were from Shen-neng Bo Cai (Shanghai,China), p-Ser and p-Thr antibodies, immunoglobulin IgG, SREBP-2 antibody, and protein A/G plus agarose were obtained from Santa Cruz Biotechnology (Santa Cruz, CA). Mouse LMNB1 monoclonal antibody was from Proteintech (ProteinTech Group, Chicago, IL, USA). [γ-^32^P]ATP (specific activity 3000Ci/mmol) was from Perkin Elmer (San Jose, CA, USA). 10×Assay buffer, Adenosine-5'-triphosphate (ATP), Acetylated-Lysine Antibody, DYKDDDDK Tag Antibody, phospho-Ser79 ACC1, anti-β-actin antibody, Rabbit polyclonal AMPK antibody and phospho-AMPK (Thr-172) antibody were purchased from Cell Signaling Technology (Beverly, MA), and rabbit polyclonal AMPK antibodies recognize the subunit α1 or α2 isoform. SAMS peptide (HMRSAMSGLHLVKRR) was purchased from Upstate Biotechnology (Lake Placid, NY). P81 phosphocellular paper was from GE healthcare (Piscataway, NJ). Trizol and Lipofectamine 2000 were purchased from Invitrogen (Carlsbad, CA).

### Cell culture and treatments

The Human normal liver cell line L-02 and HepG2 human hepatoma cell line were obtained from the Type Culture Collection of the Chinese Academy of Sciences, Shanghai, China. HepG2 cells were cultured in Eagle’s minimum essential medium (EMEM) (GIBCO) containing 10% fetal bovine serum (FBS), 100 units/ml penicillin, and 100μg/ml streptomycin, and the L-02 cells was cultured as previously described [1]. Cells were incubated in a humidified atmosphere of 5% CO_2_ at 37°C. When the cells were 80% confluent and subjected to be treated after overnight serum depletion as previously described [2,23].

### Ethics statement

The use of animals in this study was in strict accordance with the relevant federal guidelines and institutional policies. The protocol was approved by the Animal Care and Use Committee of Shandong Provincial Hospital affiliated to Shandong University (approval number: NO. 2012–090). All surgical procedures were performed under sodium pentobarbital anesthesia, and all efforts were made to minimize suffering.

### Animal experiments

Wildtype TSH receptor mice (*Tshr* ^*+/+*^ mice) and TSH receptor knockout mice (*Tshr* ^*-/-*^ mice) have been previously described [2]. *Tshr* ^*-/-*^ mice were fed a diet containing 100 ppm thyroid powder (Sigma) from 21 days-old. When fed the supplemented diet for 3 and 5 weeks, 6-week-old and 8-week-old wild-type mice and and the same age of *Tshr* ^*-/-*^ mice were collected blood to measure the level of serum TSH and total thyroxine4 (TT4), to eliminate the effect of abnormal thyroid hormone levels on metabolism, they exhibited equal levels of serum TSH and TT4. On the experimental day, 8-week-old *Tshr* ^*+/+*^ and *Tshr* ^*-/-*^ mice were divided into two groups (n = 5 per group) and were given an intraperitoneal injection of either phosphate-buffered saline (PBS) or AICAR [0.5 mg/g body wt-1] three times a week for 2 weeks (on Monday, Wednesday and Friday). PBS (control) was injected at the same volume and in the same manner as AICAR. After the treatment period, the animals were weighed and euthanized using pentobarbital sodium, and the livers were harvested and processed for subsequent analysis.

### RNA extraction and Real-Time PCR

Total RNA was extracted from cultured HepG2 cells or mouse livers using the Trizol method. First-strand cDNA was generated using a commercial PrimeScript RT reagent kit (TaKaRa, Otsu, Shiga, Japan). The resulting cDNA was amplified by real-time RT-PCR, using a SYBR Premix Ex Taq II (TaKaRa) and a LightCycler480 instrument (Roche Diagnostics). The mRNA levels of genes were normalized to that of β-actin and presented as relative to the control. The relative quantification of gene expression was analyzed by the 2^-ΔΔCt^ method [24]. The PCR primers were designed and listed in Table 1.

**Table 1:** Quantitative RT-PCR primers.

Gene	Species	Forward primer	Reverse primer
HMGCR	mouse	ATCCAGGAGCGAACCAAGAG	TACAGAAGCCCCAAGCACAA
HMGCS	mouse	AAATGCCAGACCTACAGGTGG	ATGCTGCATGTGTGTCCCA
β-actin	mouse	ACCCCAGCCATGTACGTAGC	GTGTGGGTGACCCCGTCTC
SREBP-2	human	CCCTTCAGTGCAACGGTCATTCAC	TGCCATTGGCCGTTTGTGTC
HMGCR	human	CTTGTGTGTCCTTGGTATTAGAGC	ATCATCTTGACCCTCTGAGTTACAG
HMGCS	human	CATTAGACCGCTGCTATTCTGTC	TTCAGCAACATCCGAGCTAGA
β-actin	human	ACAGAGCCTCGCCTTTGCCG	ACATGCCGGAGCCGTTGTCG

### Protein extraction and Western blotting

Nuclear and cytoplasmic extracts were prepared using the NE-PER nuclear and cytoplasmic extraction reagent kit (Pierce Biotechnology Inc.) according to the manufacturer's instructions. Total protein extract and Western blotting (WB) analysis were conducted as described previously [2].

### Immunoprecipitation

The immunoprecipitation of the specific proteins from HepG2 cells was carried out as previously described [25].

### AMPK activity assay

AMPK activity was measured using the SAMS peptide assay. Total protein (500 μg) was immunoprecipitated with polyclonal rabbit AMPKα antibodies as previously described [23].

### Plasmid and transfection

The expression vector pcDNA3.1–2×FLAG SREBP-2 (human, amino acids 1–481) was obtained from Addgene (Plasmid 26807,provided by Timothy Osbome, La Jolla, CA) [26]. For transient expression assays, HepG2 cells were cultured in complete medium with 10% FBS to -80% cell confluence, synchronized overnight in serum-free EMEM in 6cm dishes and then transfected with the plasmids using Lipofectamine 2000 according to the manufacturer’s protocols.

### Immunofluorescence staining

SREBP-2 immunofluorescent staining in HepG2 cells was performed to determine the subcellular localization of SREBP-2. HepG2 cells seeded onto cover slides were treated without or with AICAR (0.5 mM) for 12 or 24 h. Immunofluorescence was then performed. Cover slides were incubated overnight at 4°C with an SREBP-2 antibody and subsequently with a secondary antibody (conjugated to TRITC); the nuclei were stained with 4’6-diamidino-2-phenylindole (DAPI). Fluorescence was visualized on Karl Zeiss A2 microscopes.

### Double Immunofluorescence Staining for SREBP-2 and phospho-Thr antibody in HepG2 cells

Because specific antibodies to detect threonine–phosphorylated SREBP-2 were not available, we used double immunofluorescence staining with SREBP-2 and phospho-Thr antibody. Staining was carried out as described [27].

### Statistical analysis

Mean ± SEM values were analyzed using GraphPad Prism (version 5; GraphPad Software Inc, San Diego, CA). All of the experiments were repeated at least three independent times. We assessed the differences between means by one-way analysis of variance followed by Tukey’s multiple comparison tests. Differences were considered significant at p < 0.05.

## Results

### TSH increased the expression of SREBP-2, HMGCR and HMGCS in HepG2 cells.

**Fig 1:**
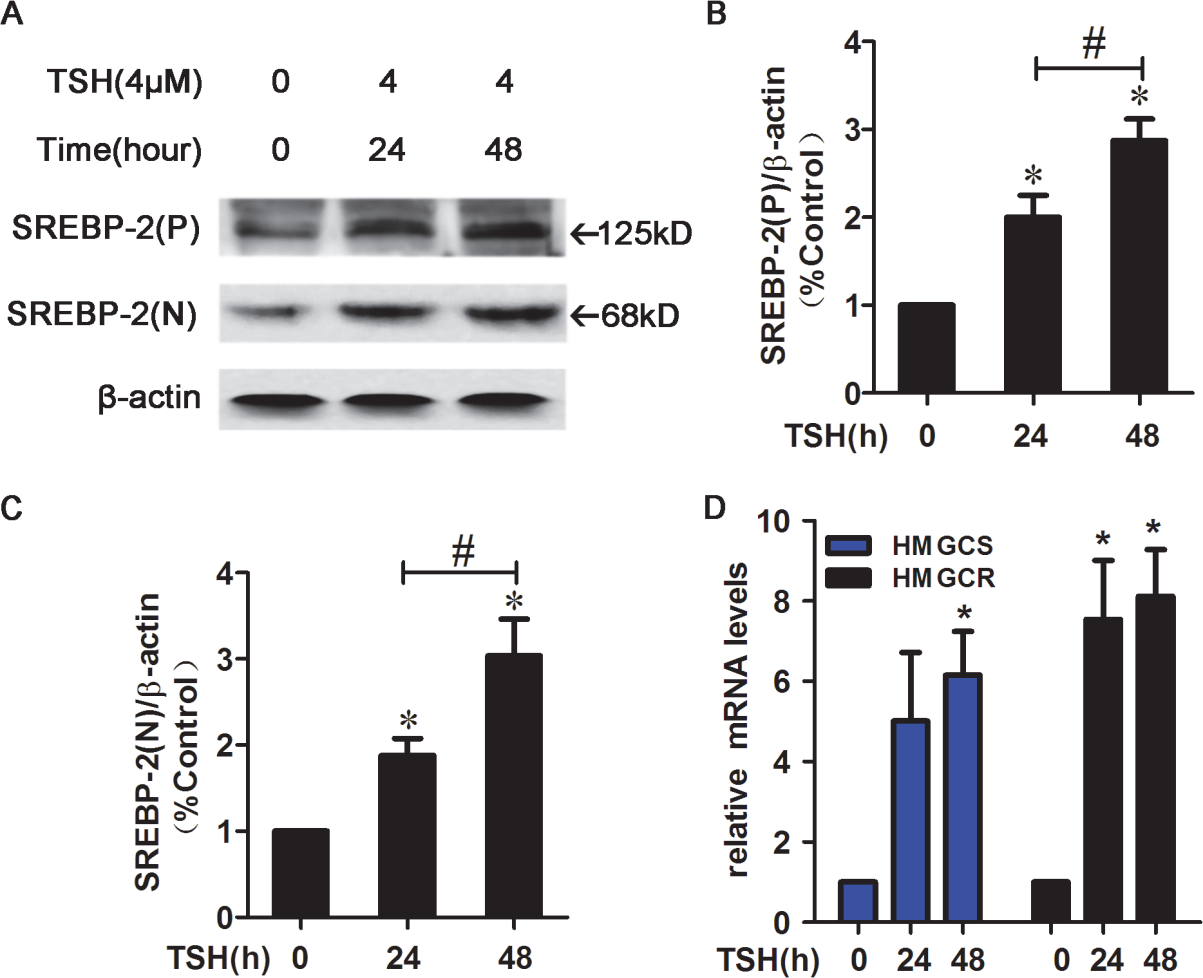
TSH increased SREBP-2 protein levels and the expression of its target genes, HMGCR and HMGCS, in HepG2 cells. (A) HepG2 cells were pretreated with TSH (4 μM) for 24 or 48 h. Whole cell lysates were subjected to Western blotting (WB) using an SREBP-2 antibody that recognizes both the SREBP-2 precursor and nuclear active forms. (P) and (N) denote the precursor and nuclear active forms of SREBP-2, respectively. (B-C) Densitometric quantifications of SREBP-2 (P) and SREBP-2 (N) are shown. Densitometry was performed using ImageJ (version 1.45) and normalized to β-actin. The data are presented as the mean ± SEM. *p< 0.05 versus zero concentration of TSH, ^#^ *p* < 0.05 versus TSH (24h). (D) HepG2 cells were treated with TSH (4 μM) for 24 or 48 h and then were harvested to monitor the mRNA expression of HMGCR and HMGCS. β-actin was used for normalization, and the control was set to 1 in the Real-Time PCR data. All the experiments were performed in duplicate. *p < 0.05 versus zero concentration of TSH.

SREBP-2 preferentially regulates genes that are responsible for cholesterol synthesis and uptake in the liver. The protein levels of the SREBP-2 precursor and the nuclear active form increased in a time-dependent manner in HepG2 cells after treatment with TSH (4 μM) (Fig 1A–1C). Because we previously determined that TSH did not significantly affect LDLR expression [1], we focused on the two rate-controlling enzymes, HMGCR and HMGCS. As expected, TSH treatment up-regulated HMGCS mRNA expression at 48 h and HMGCR mRNA expression at both 24 and 48 h (*p*<0.05) (Fig 1D).

### AMPK activation by AICAR directly inhibited SREBP-2 expression in HepG2 cells.

**Fig 2:**
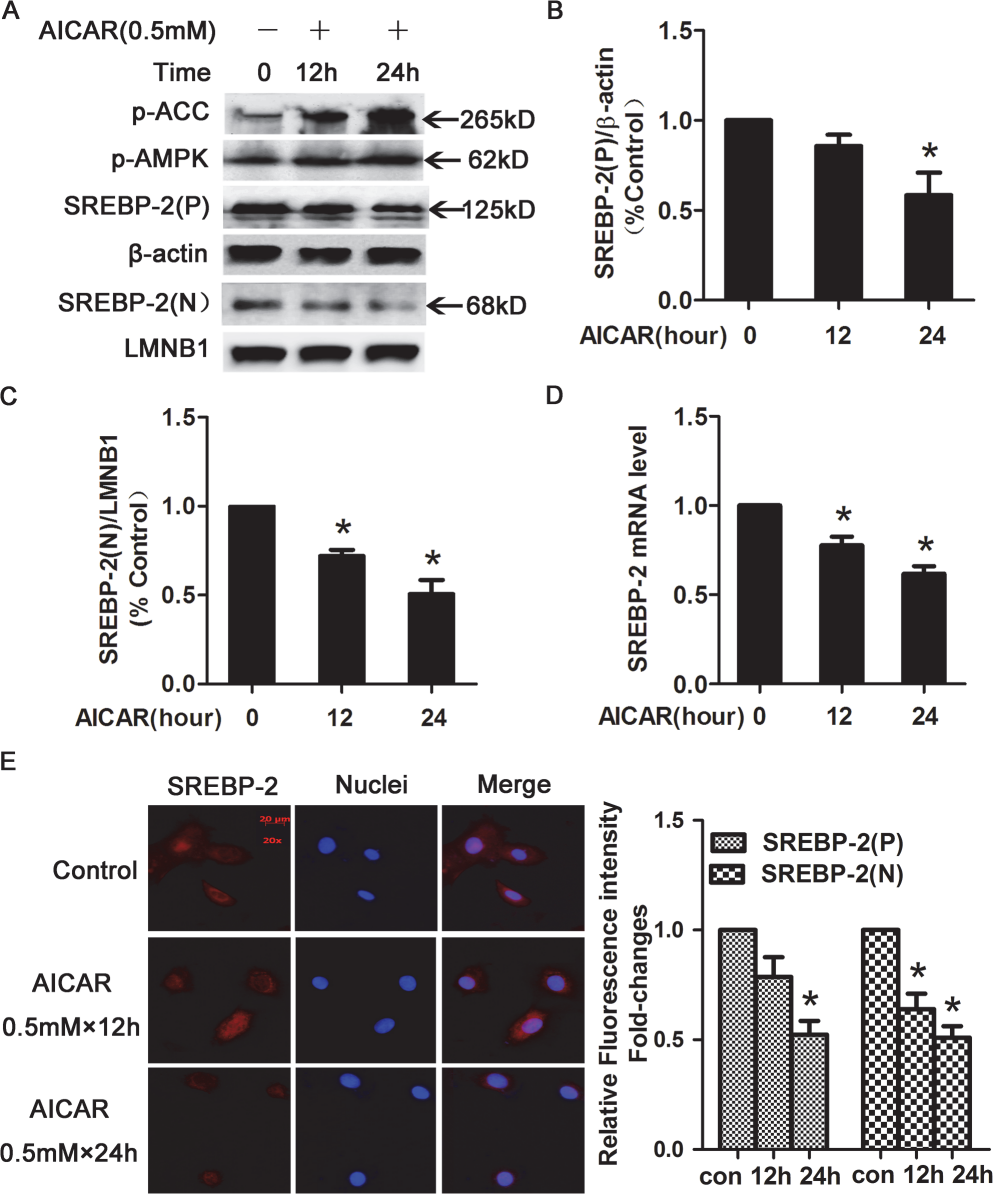
AICAR-activated AMPK decreased SREBP-2 protein levels. (A) HepG2 cells were treated with or without 0.5 mmol/l (0.5 mM) AICAR for 12 or 24 h and were subsequently washed with phosphate-buffered saline (PBS) and lysed. Nuclear and cytoplasmic extracts were analyzed by WB. β-actin was used as a cytoplasmic marker, and LaminB1 (LMNB1) was used as the nuclear marker. (B-C) Densitometric quantification of SREBP-2 (P) and SREBP-2 (N). The data are presented as the mean ± SEM. *p< 0.05 versus control (con, without AICAR). (D) AICAR-activated AMPK inhibited the expression of SREBP-2 in HepG2 cells. HepG2 cells were treated with or without 0.5 mM AICAR for 12 or 24 h and then were harvested to determine the mRNA expression of SREBP-2. β-actin was used for normalization, and the control was set to 1 in the Real-Time PCR data. All the experiments were performed in duplicate. *p < 0.05 versus control. (E) Immunofluoresence images of SREBP-2 (red) and nuclear staining with 4', 6-diamidino-2-phenylindole (DAPI, blue) in HepG2 cells. Magnification, ×200. Semiquantification analysis by ImageJ software of fluorescence intensity of SREBP-2 precusor and nuclear form in HepG2 cells. Bar-graph represents the results from 3 separate experiments and the fluorescence intensity of SREBP-2 from HepG2 cells without AICAR treatment was set as 1.

SREBP-2 is regulated in multiple ways, including sterol feedback mechanisms and modulation of mRNA expression. SREBP-2 is a direct target of AMPK, which inhibits SREBP-2 transcriptional activity [22]. To explore whether AMPK activation regulates the expression of the SREBP-2 precursor and nuclear active forms, we treated HepG2 cells with AICAR (an AMPK activator) for 12 or 24 h. Phospho-AMPK (p-AMPK) and phospho-ACC (p-ACC), indicators of AMPK activation [17], were markedly up-regulated in a time-dependent manner (Fig 2A). The SREBP-2 precursor levels slightly decreased in response to AICAR at 12 h and were significantly decreased by 24 h (Fig 2B). However, the nuclear active form of SREBP-2 exhibited a significant decrease during the AICAR treatment period (Fig 2C).

To confirm the data above, SREBP-2 mRNA expression was determined by real-time PCR. AICAR-mediated AMPK activation decreased SREBP-2 mRNA expression in a time-dependent manner (Fig 2D). Similar results are shown in Fig 2E, which illustrates that the immunofluorescent staining intensity of nuclear SREBP-2 was obviously reduced by AICAR. These data indicated that AMPK activation directly inhibited SREBP-2 expression and activity.

### AMPK activation reduced SREBP-2 protein levels and target gene expression in the livers of AICAR-treated *Tshr* ^*-/-*^ and *Tshr* ^*+/+*^ mice.

**Fig 3:**
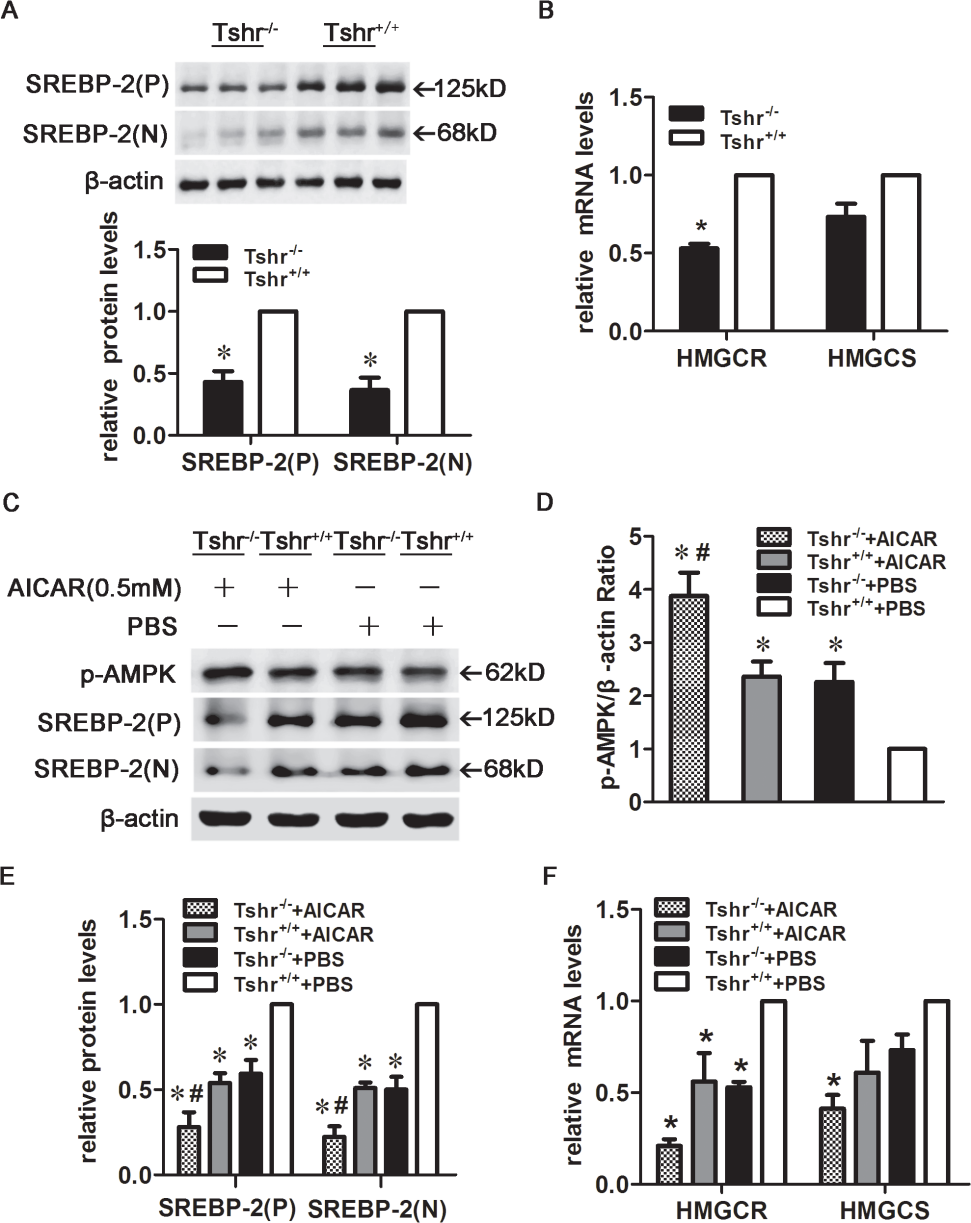
AMPK activation reduced SREBP-2 protein and target gene expression in the livers of AICAR-treated *Tshr* ^*-/-*^ and *Tshr* ^*+/+*^ mice. (A) SREBP-2 protein levels were decreased in *Tshr* ^*-/-*^ mice (n = 3 animals/group), Densitometric quantifications of SREBP-2 (P) and SREBP-2 (N) are shown. (B) HMGCR and HMGCS expression in the livers of *Tshr* ^*-/-*^ and *Tshr* ^*+/+*^ mice. *p < 0.05 versus *Tshr* ^*+/+*^ mice. (C) AMPK activation decreased the expression of the SREBP-2 precursor and nuclear forms in the livers of AICAR-treated *Tshr* ^*-/-*^ and *Tshr* ^*+/+*^ mice. *Tshr* ^*-/-*^ and *Tshr* ^*+/+*^ mice were injected intraperitoneally three times a week for 2 weeks with AICAR or PBS, and liver extracts were subjected to WB with antibodies against p-AMPK and SREBP-2. The WBs are representative of the 5 animals per group. (D-E) Densitometric quantifications of p-AMPK, SREBP-2 (P) and SREBP-2 (N). The data are presented as the mean ± SEM. *p< 0.05 versus PBS-treated *Tshr* ^*+/+*^ mice, ^#^ *p* < 0.05 versus PBS-treated *Tshr* ^*-/-*^ mice. (F) HMGCR and HMGCS expression in the livers of AICAR-treated *Tshr* ^*-/-*^ and *Tshr* ^*+/+*^ mice. The data are presented as the mean ± SEM. *p < 0.05 versus PBS-treated *Tshr* ^*+/+*^ mice.

It has been previously reported that TSH suppresses AMPK activity in the thyroid gland and the liver [2, 28]. To further investigate the effects of TSH and AMPK on SREBP-2, we established *Tshr* ^*+/+*^ and *Tshr* ^*-/-*^ mouse models. As shown in Fig 3A, the SREBP-2 precursor and nuclear active forms were decreased in the livers of *Tshr* ^*-/-*^ mice compared with *Tshr* ^*+/+*^ mice, although the exact mechanisms by which this occurs remain unclear. The mRNA expression of HMGCR was also significantly reduced, and HMGCS mRNA expression exhibited a decreasing trend (Fig 3B).

AMPK activity increased in the livers of PBS-treated *Tshr* ^*-/-*^ mice compared with *Tshr* ^*+/+*^ mice. Furthermore, the SREBP-2 precursor and nuclear active forms were significantly down-regulated in *Tshr* ^*-/-*^ mice. AICAR treatment enhanced AMPK activity and reduced SREBP-2 protein levels in the livers of both *Tshr* ^*-/-*^ and *Tshr* ^*+/+*^ mice (Fig 3C–3E). AICAR significantly decreased HMGCR mRNA expression (Fig 3F). These results indicated that AMPK activation ameliorated the SREBP-2 up-regulation induced by TSH.

### AICAR stimulated AMPK activity and suppressed the TSH-induced up-regulation of SREBP-2 in HepG2 cells.

**Fig 4:**
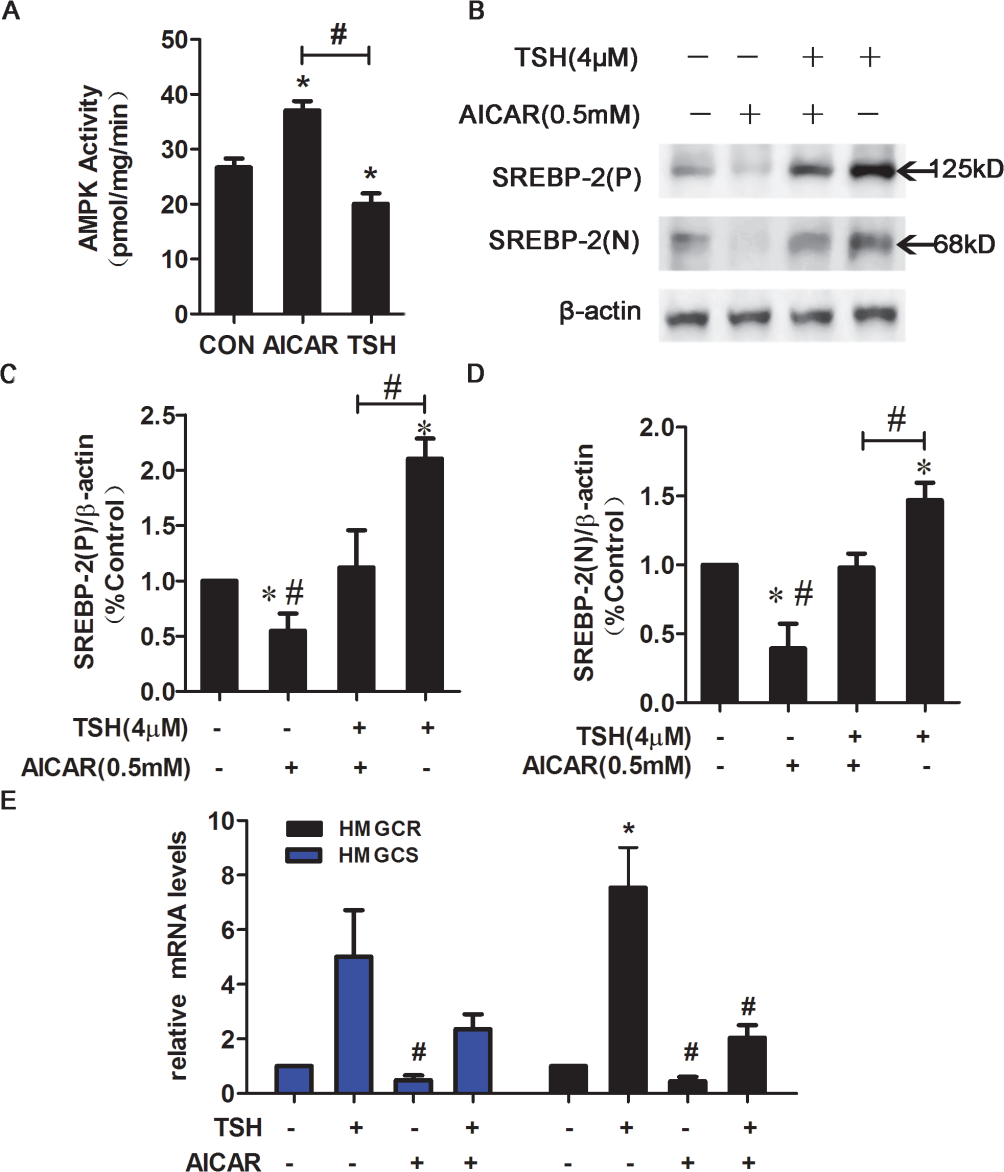
AMPK activation by AICAR attenuated the TSH-mediated increase in SREBP-2. (A) TSH decreased AMPK activity. HepG2 cells were synchronized overnight in serum-free EMEM and then treated with AICAR (0.5 mM) or TSH (4 μmol/L) for 24 h. AMPK activity was measured using the SAMS peptide phosphorylation assay and was calculated as picomoles per minute per milligram protein. The data are presented as the mean ± SEM (n = 4). *p < 0.05 compared with control (con). ^#^ *p* < 0.05 versus TSH. (B) HepG2 cells were pretreated with TSH (4 μM) for 24 h in the absence or presence of AICAR (0.5 mM) for 24 h. Whole cell lysates were subjected to WB using the SREBP-2 antibody. (C-D) Densitometric quantification of SREBP-2 (P) and SREBP-2 (N). The data are presented as the mean ± SEM. *p < 0.05 versus untreated cells; ^#^ *p* < 0.05 versus TSH-treated cells. (E) AICAR activated AMPK, which down-regulated the mRNA expression of HMGCR and HMGCS in TSH-treated HepG2 cells. These experiments were repeated at least three times with similar results.

To further confirm the effects of TSH on AMPK activity, we performed a SAMS peptide phosphorylation assay. In HepG2 cells, AMPK activity was markedly decreased by TSH compared with control and increased by AICAR treatment (Fig 4A). We next examined whether the TSH-induced up-regulation of SREBP-2 occurred via AMPK activation. As shown in Fig 4B–4D, the TSH-induced up-regulation of the SREBP-2 precursor and nuclear active forms was ameliorated by AICAR (Fig 4B–4D).

To determine the functional consequences of the AMPK-mediated suppression of SREBP-2, the gene expression of SREBP-2 target genes was assessed by RT-PCR. Consistent with the suppressed SREBP-2 protein levels, the TSH-mediated up-regulation of HMGCR and HMGCS mRNA expression was attenuated by AICAR in HepG2 cells (Fig 4E). These data indicated that AICAR activated AMPK and suppressed the TSH-induced up-regulation of SREBP-2 as well as SREBP-2 target gene transcription.

### Threonine phosphorylation of SREBP-2 was observed in AICAR-treated cells with activated AMPK and in mouse livers.

**Fig 5:**
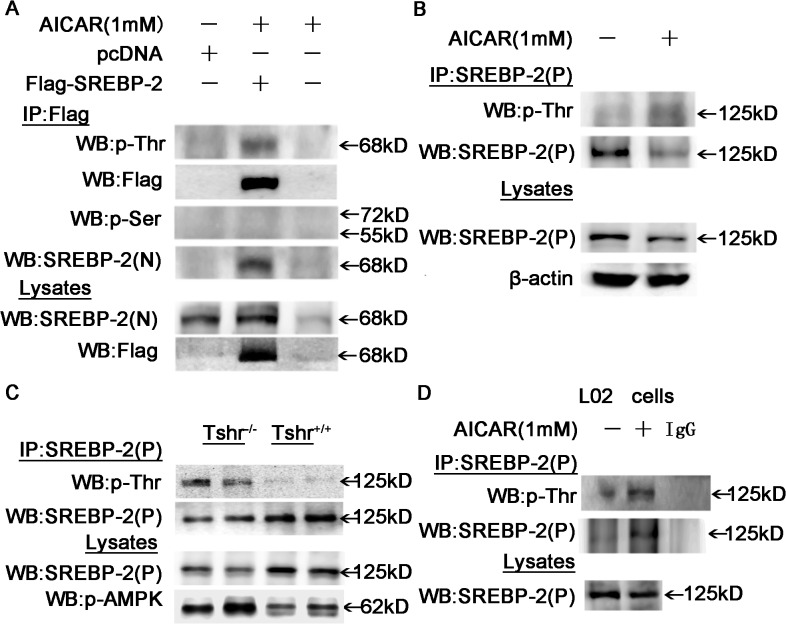
AMPK activation by AICAR induced the threonine phosphorylation of the precursor and nuclear forms of SREBP-2. (A) HepG2 cells were transfected with pc-DNA3.1 encoding 2×flag-tagged human nuclear SREBP-2 or pcDNA empty vector or vehicle for 48 h and then were treated with or without AICAR (1 mM) for 24 h. Cell lysates were purified by immunoprecipitation (IP) with an anti-Flag antibody and were subjected to WB with antibodies against phosphorylated-Threonine (p-Thr) or phosphorylated-Serine (p-Ser). Total lysates were analyzed by WB with anti-flag and SREBP-2 antibodies as indicated. (B) HepG2 cells were treated with AICAR (1 mM) for 24 h, and the cell lysates were immunoprecipitated with an SREBP-2 antibody and subjected to WB with antibodies against p-Thr and p-Ser (data not shown). SREBP-2(P) denotes the antibody that only recognizes the SREBP-2 precursor. (C) Total protein (500 μg) from *Tshr* ^*+/+*^ and *Tshr* ^*-/-*^ mouse liver extracts were purified by IP with the SREBP-2 precursor antibody and subjected to WB with the p-Thr antibody. Total lysates were analyzed by WB with antibodies against p-AMPK and SREBP-2 as indicated. (D) Lysates from L02 cells treated with AICAR for 24 h were subjected to IP with an SREBP-2 antibody and then analyzed by WB with an antibody against p-Thr.

AMPK, an evolutionally conserved serine/threonine kinase, plays an important role in regulating key metabolic processes by phosphorylating multiple enzymes involved in fatty acid oxidation and cholesterol synthesis, leading to their activation or inhibition. A recent study demonstrated that SREBP-2 is a direct target of AMPK in HEK293T cells and in the *LDLR* ^-/-^ mouse liver [22]. To further investigate whether AMPK is involved in the phosphorylation of SREBP-2, we detected phosphorylation at threonine (p-Thr) and serine (p-Ser) residues with specific antibodies. Immunoprecipitation of total protein with SREBP-2 antibodies and immunoblot analysis with p-Ser or p-Thr antibodies demonstrated that the SREBP-2 precursor and nuclear forms were phosphorylated on threonine residues, not on serine residues, in HepG2 cells (Fig 5A and 5B). AMPK activity was higher in the livers of *Tshr* ^-/-^ mice than in *Tshr* ^*+/+*^ mice, and consistent with the above observations, the level of threonine phosphorylation of the endogenous SREBP-2 precursor was higher in *Tshr* ^-/-^ mice than in *Tshr* ^*+/+*^ mice (Fig 5C). Similar results are obtained using liver L02 cells (Fig 5D). These results demonstrated a direct interaction between AMPK and SREBP-2 that involved protein phosphorylation.

### TSH repressed the AICAR-mediated phosphorylation of SREBP-2.

**Fig 6:**
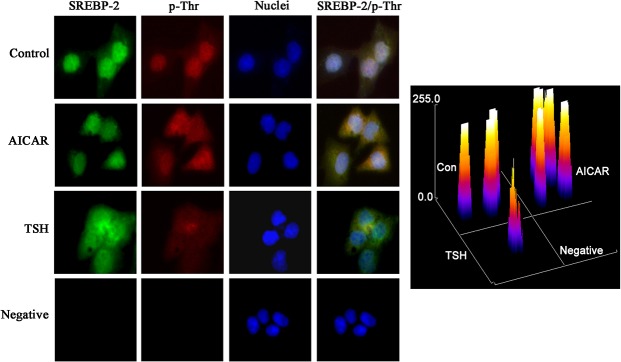
Double immunofluorescence staining for phosphorylated SREBP-2 in HepG2 cells. HepG2 cells were treated with AICAR (1 mmol/L) for 12 h or TSH (4 μM) for 24 h. The yellow in the images is derived from dual staining with the red p-Thr antibody and the green anti-SREBP-2 antibody. The nuclei were detected with 4', 6-diamidino-2-phenylindole (DAPI). Original magnification, The intensity of staining obtained with SREBP-2/p-Thr was measured by ImageJ.

As mentioned above, TSH suppresses AMPK activity in the liver; therefore, we hypothesized that TSH could influence the AMPK-induced phosphorylation of SREBP-2. Because specific phosphorylated SREBP-2 antibodies were not available, we utilized double immunofluorescence staining to test this hypothesis. As shown in Fig 6, phosphorylated threonine was visualized as red, and SREBP-2 was visualized as green. The colocalization of phosphorylated threonine and SREBP-2 (phosphorylated-SREBP-2) produced a yellow signal. AICAR treatment significantly increased the levels of phosphorylated SREBP-2 (yellow), but this effect was inhibited by TSH.

## Discussion

**Fig 7:**
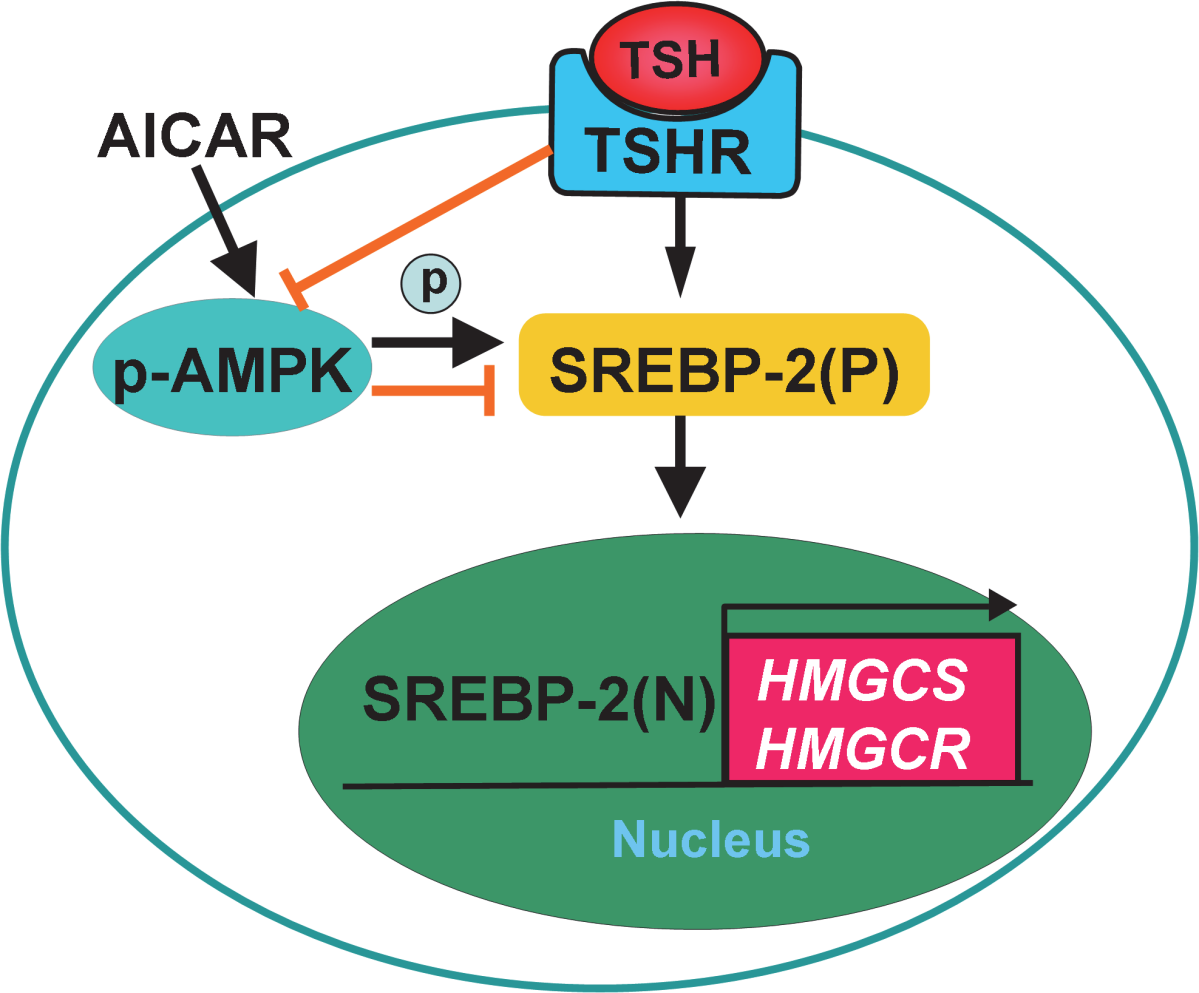
Proposed regulatory mechanism of SREBP-2 by TSH and AMPK.

The present study demonstrated that AICAR activated AMPK, which directly phosphorylated SREBP-2 at threonine residues and suppressed its up-regulation by TSH, thereby leading to decreased transcription of SREBP-2 and its target genes (Fig 7 **)**. These data may represent a molecular mechanism by which AMPK activation ameliorates hepatic steatosis and hypercholesterolemia associated with high TSH levels in SCH patients.

It has long been known that thyroid hormones play an important role in lipid metabolism, dyslipidemia is a common finding in patients with clinical hypothyroidism, including mainly elevated levels of total and LDL cholesterol, the underlying mechanism is widely thought to be thyroid hormones deficiency [7]. Subclinical hypothyroidism (SCH), which is characterized by normal thyroid hormone levels and elevated TSH levels [7], is a considerably more common metabolic disorder than clinical hypothyroidism. Patients with SCH are usually asymptomatic, but because of its association with dyslipidemia, these patients typically present with increased total and LDL cholesterol; therefore, SCH is as an independent risk factor for atherosclerosis as well as obesity, hypertension, and diabetes [3, 29, 30]. But it is hard to explain hypercholesterolemia in SCH only by the role of thyroid hormone, recent studies have focused on the relationship between TSH and lipids, aiming to elucidate the details of this association [1, 3, 4, 6] and suggesting that inhibiting TSH may be important for preventing dyslipidemia and hepatic steatosis.

Our previous studies demonstrated that TSH up-regulates hepatic HMGCR and SREBP-2 expression (unpublished) in the liver [1]. SREBP-2 is a key transcription factor that regulates HMGCR and HMGCS expression. In the present study, the SREBP-2 precursor and nuclear active forms as well as the HMGCR and HMGCS target genes were significantly up-regulated by TSH in HepG2 cells, which was consistent with our previous findings. Interestingly, this up-regulation was suppressed by AMPK after activation with AICAR. We confirmed this observation in vivo using knockout mice. *Tshr* ^*-/-*^ mice exhibited increased AMPK activity and decreased expression of HMGCR, HMGCS, and the SREBP-2 precursor and nuclear active forms in the liver compared with *Tshr* ^*+/+*^ mice.

AMPK is a crucial cellular and whole-body energy sensor that phosphorylates and regulates numerous proteins involved in nutrient metabolism and thereby has a pleiotropic beneficial effect in different tissues. Recently, AMPK has attracted considerable interest as a therapeutic target to treat the metabolic dysfunction observed in Metabolic Syndrome and diabetes [18]. AMPK decreases lipogenesis in the liver, mainly by phosphorylating and inactivating ACC1 and HMGCR, thereby inhibiting de novo fatty acid and cholesterol synthesis. Li and colleagues demonstrated that AMPK interacts with and phosphorylates SREBP-1c and SREBP-2, inhibiting the SREBP activation steps of proteolytic maturation and nuclear translocation [22]. In our study, after activation by AICAR, AMPK directly phosphorylated threonine residues in SREBP-2, and the SREBP-2 precursor and the nuclear active form of SREBP-2 in particular were significantly down-regulated in a time-dependent manner; these results are consistent with the data published by Li and others [31, 32]. The down-regulation of SREBP-2 was accompanied by a significant decrease in HMGCR mRNA expression and a slight decrease in HMGCS mRNA expression; this disparity may derive from the differential effects of SREBP-2 in stimulating target gene transcription. Our results indicate that AMPK activation by AICAR could suppress up-regulation of SREBP-2 induced by TSH; on the other hand, AMPK activation by AICAR alone also reduced SREBP-2 protein levels in HepG2 cells and both Tshr^+/+^ and Tshr^-/-^ mice, indicating that the effect of AMPK on the SREBP-2 pathway is independent of TSH stimulation (Fig 7).

We and other researchers have reported that TSH suppresses AMPK phosphorylation/activation in the thyroid gland and liver [2, 28]. Although the precise mechanism for the inhibition of AMPK by TSH has not yet been fully elucidated, we determined that TSH interacted with AMPK to influence the phosphorylation of SREBP-2, but the biological implications of SREBP-2 phosphorylation are complex. It has been demonstrated that the nuclear form of SREBP-2 is modified by phosphorylation [25, 33], acetylation [34], sumoylation [35], and ubiquitination [36], and these modifications interact and regulate SREBP-2 stability and/or transcriptional activity. Ser432/Ser436 can be phosphorylated in nuclear SREBP-2, which enhances its ubiquitination and proteasome-mediated degradation through a phosphorylation-dependent mechanism [36]. We speculate that the phosphorylation of SREBP-2 at threonine residues may significantly enhance its degradation, consequently leading to the reduced expression of SREBP-2-regulated genes. Future studies are necessary to identify the specific phosphorylation site to fully understand the mechanisms of SREBP-2 regulation.

In conclusion, the current study demonstrated that after activation by AICAR, AMPK directly phosphorylated SREBP-2 at threonine residues and suppressed its up-regulation by TSH. The pharmacological stimulation of AMPK may have considerable potential for reversing the dyslipidemia associated with high TSH levels in SCH patients.
